# Occupational Therapy in Complex Patients: A Pilot Randomized Controlled Trial

**DOI:** 10.1155/2018/3081094

**Published:** 2018-09-03

**Authors:** Martina Pellegrini, Debora Formisano, Veronica Bucciarelli, Margherita Schiavi, Stefania Fugazzaro, Stefania Costi

**Affiliations:** ^1^Department of Physical and Rehabilitation Medicine, Arcispedale Santa Maria Nuova-IRCCS, Azienda Unità Sanitaria Locale, 42100 Reggio Emilia, Italy; ^2^Scientific Directorate, Arcispedale Santa Maria Nuova-IRCCS, Azienda Unità Sanitaria Locale, 42100 Reggio Emilia, Italy; ^3^BSc in Occupational Therapy, Azienda Unità Sanitaria Locale, 42100 Reggio Emilia, Italy; ^4^Children Rehabilitation Special Unit, Arcispedale Santa Maria Nuova-IRCCS, Azienda Unità Sanitaria Locale, 42100 Reggio Emilia, Italy; ^5^Surgical, Medical and Dental Department of Morphological Sciences related to Transplant, Oncology and Regenerative Medicine, Università degli Studi di Modena e Reggio Emilia, 41100 Modena, Italy

## Abstract

**Introduction:**

To determine effect size and feasibility of experimental occupational therapy (OT) intervention in addition to standard care in a population of complex patients undergoing rehabilitation in a hospital-home-based setting.

**Method:**

40 complex patients admitted to the rehabilitation ward of the Local Health Authority-Research Institute of Reggio Emilia (Italy) were randomized in a parallel-group, open-label controlled trial. Experimental OT targeting occupational needs in the areas of self-care, productivity, and leisure was delivered by occupational therapists. Standard care consisted of task-oriented rehabilitation delivered by a multiprofessional team.

**Results:**

The experimental OT intervention was completed by 75% of patients assigned to this group. The average changes in the Canadian Occupational Performance Measure (COPM) performance score significantly and clinically favored experimental OT [−3,06 (−4.50; −1.61); delta > 2 points, resp.]. Similar trends were detected for COPM satisfaction and independence in instrumental activities of daily living (ADL). At follow-up, level of social participation was higher for patients treated with experimental OT (*p* = 0.043) than for controls.

**Conclusions:**

Experimental OT was feasible in complex patients in a hospital-home-based setting. It ameliorated both patients' performance and satisfaction in carrying out relevant activities and improved independence in instrumental ADL. The trial is registered with ClinicalTrials.gov NCT02677766.

## 1. Introduction

Since the introduction of the International Classification of Functioning, Disability and Health (ICF) in 2001, the World Health Organization has been promoting the implementation of a client-centered, biopsychosocial approach in healthcare programs and rehabilitation services [1]. This conceptual framework focuses on individual functioning more than on disease and contemplates the health condition as a dynamic status resulting from a comprehensive view of biological, individual, and social perspectives [1].

The ICF approach is particularly appropriate for patients during rehabilitation, as the latter relies on the interaction between functions, activities, participation, and contextual factors [2, 3]. Indeed, regardless of the underlying pathology, patients undergoing rehabilitation habitually manifest similar basic needs, while their level of functioning and advanced needs, namely, those related to leisure, productivity, and social role, may be different [4]. This was brought to light also by Phipps and Richardson's work [5], which showed that when rehabilitation focuses on individual significant activities, gains in performance and satisfaction are noticeable both in patients with traumatic brain injury and in those with stroke. Similarly, occupational therapy (OT) has proven to be beneficial in patients with different types of cancer when based on patient requests [6].

In line with this approach, an assessment tool based on the complexity of patients' care needs has been validated to classify individuals who require rehabilitation interventions [7, 8]. This classification makes it possible to create homogeneous populations according to the complexity of their care needs, not to their diagnosis. Moreover, this approach may facilitate conducting valid rehabilitative studies, whose results may be highly generalizable.

A complex patient suffers from a disease that affects clinical stability and functional autonomy. This patient is dependent on others when carrying out daily activities and manifests regular need for medical monitoring, for specialized nursing care and for two or more specialized interventions, for example, occupational therapy, physiotherapy, or speech therapy. Frequently, complex patients need special aids to carry out tasks. Thus, complex patients benefit from multiprofessional rehabilitation, which may include client-centered OT interventions.

Our research group recently conducted an observational study aimed at identifying the needs of complex inpatients in a rehabilitation ward to develop a client-centered OT intervention targeted at this population [9]. To our knowledge, no well-designed randomized clinical trial on the efficacy of OT in the rehabilitation process of complex patients has yet been published.

We thus decided to conduct this ICF concept-based pilot randomized controlled trial (RCT) in order to detect the effect size of an experimental, client-centered OT intervention in a population of complex patients in their rehabilitation phase.

## 2. Materials and Methods

This single-center, open-label RCT with two parallel groups was designed in accordance with the CONSORT statement and the Helsinki declaration. The study was approved by the local Ethics Committee (19/03/2014, number 325).

### 2.1. Study Objectives

The primary aim of this exploratory study was to estimate the effects of experimental OT on complex patients' perception of occupational performance during relevant activities. If the experimental OT proved beneficial, its effect size estimate would be used to plan a powered randomized controlled trial.

The secondary aim was to verify the feasibility of the OT experimental intervention in a mixed hospital-home-based setting for a population of complex patients undergoing rehabilitation.

Further objectives were to estimate the effects of experimental OT on (a) complex patients' self-perception of occupational satisfaction with the way they perform their relevant occupational activities, (b) mood disturbances, (c) independence in basic and instrumental activity of daily living (ADL), and (d) reintegration to normal social activities and quality of life (QoL).

### 2.2. Participants

All adult patients admitted to the Physical and Rehabilitation Medicine (PRM) ward of the Local Health Authority-Research Institute (AUSL-IRCCS) of Reggio Emilia, Italy, and deemed complex on the basis of the Rehabilitation Complexity Scale-Extended (RCS-E) were screened for eligibility. The RCS-E score ranges from zero to 22, with the cut-off value for complexity set at nine [7].

Exclusion criteria were the presence of severe cognitive impairment, verified by the physiatrist through direct observation and an exploratory interview (evaluating memory, orientation in time and space, adequacy to the context, absence of disinhibition or frontal disorders, and risk evaluation), primary psychiatric disorders, communication disability, and language barriers that, in the opinion of the healthcare team, would prevent the patient from participating in the experimental OT program. Furthermore, to allow assessment of the feasibility of the experimental intervention, patients living over 30 km from the hospital and patients for whom it was known a priori that they would be discharged to a retirement home were excluded.

We also excluded complex patients already recruited in a competing clinical trial (ISRCTN75290225).

Informed consent was obtained from all participants by physicians during the admission process.

### 2.3. Outcomes

The primary outcome measure for this study was the performance score of the Canadian Occupational Performance Measure (COPM) [10]. The COPM is a standardized client-centered measure designed to detect changes in occupational performance and satisfaction over time, based on patient perception. The COPM is administered by a semistructured interview resulting in a list of up to five priority occupational activities, suited to satisfy relevant needs in three areas: self-care, productivity, and leisure.

The feasibility of the experimental OT intervention was assessed by calculating the ratio between the number of patients who completed it according to the predefined posology and the total number of patients enrolled in the intervention group (IG). We established a priori that the study would be judged feasible if 75% of patients randomized to the intervention group completed the experimental OT. Given the complex nature of these patients, we also collected information on the appropriateness of estimates made a priori regarding the timing of achievement of treatment goals and the level of independence achieved by any participant enrolled in the IG.

Further outcome measures applied to verify the effects of experimental OT in complex patients were the satisfaction score of the COPM [10], the Hospital Anxiety and Depression Scale (HADS) [11], the modified Barthel Index (MBI) [12], the Instrumental Activity Daily Living (IADL) scale [13], the Reintegration to Normal Living Index (RNLI) [14], and the Short-Form 12 (SF-12) [15].

### 2.4. Assessments

Study participants were assessed at baseline (T0), upon discharge (T1), and at follow-up (T2) (Table 1).

Baseline assessment (T0) was carried out within 1 week from admission to the PRM ward and before randomization. At T0, the degree of comorbidity was assessed using the Charlson Comorbidity Index (CCI), which is also a measure of burden of disease [16]. T0 also included the assessments of occupational performance and satisfaction, mood disturbances, B-ADL, and I-ADL.

T1 took place within three days before discharge from the PRM ward. Except for comorbidity assessment, it included all the above-mentioned measurements plus the RNLI and QoL assessments.

The follow-up (T2) took place at the patient's domicile 45 ± 15 days from discharge and included all the assessments administered at T1.

T2 assessments and all the COPM interviews were collected by the occupational therapists. Given the aim of this study, the rehabilitation team was integrated with two occupational therapists working specifically on this trial. T0 and T1 assessments were collected by members of the rehabilitation health care team (physiatrists, physiotherapists, occupational therapists, and nurses), as per habit of the ward.

Data regarding the feasibility of the experimental OT intervention in this specific hospital-home-based setting were collected by researchers throughout the trial and were unified at T2.

### 2.5. Randomization

Shortly after T0, patients were randomly assigned to the control group (CG) or to the IG, with a 1 : 1 allocation ratio. The Research and Statistics unit of the AUSL-IRCCS generated the computerized random allocation lists and proceeded with the concealed allocation of patients to groups, once the patients had been enrolled by clinicians and after T0 assessment.

Patients assigned to CG were provided with the standard care already in place in the PRM ward. Patients assigned to IG followed the experimental OT intervention delivered in addition to standard care.

### 2.6. Control Group

The CG underwent standard care, which consisted of task-oriented rehabilitation targeted at the recovery of autonomy in B-ADL (basic activity of daily life). Patients were cared for by an interdisciplinary multiprofessional rehabilitation team composed of physiatrists, nurses, physiotherapists, and speech therapists, as well as by a psychologist and social worker when necessary.

During the postacute phase, standard care was carried out daily, six days a week, during hospitalization. Standard care also included some (one to three) therapeutic authorizations to go home for the weekend in the predischarge phase. On the basis of predischarge assessment, rehabilitation was continued postdischarge on an outpatient basis when deemed necessary by the rehabilitation team.

### 2.7. Intervention Group

The experimental OT intervention was provided by the occupational therapists in addition to standard care and was based on the Canadian Model of Occupational Performance and Engagement [17]. Experimental OT is aimed at satisfying the occupational needs in the areas of self-care, productivity, and leisure that emerged through the COPM assessment at baseline.

During the postacute phase, experimental OT was delivered daily, five days a week, during hospitalization. In this setting, experimental OT was targeted at the accomplishment of occupational needs in the self-care area and, when needed, in the productivity and leisure areas.

After discharge from the PRM ward, experimental OT was delivered at the patient's domicile for up to ten sessions over a period of one to two months. In this setting, experimental OT was targeted at the accomplishment of occupational needs related to productivity and leisure areas and to any residual goals of the self-care area.

The experimental OT intervention was planned by the occupational therapists, was tailored to each patient, and was carried out according to the following phases:

1. Identification of three to five subjective priority occupational needs, which become the focus of the experimental OT intervention
2. Observation of patient while performing the activities related to the occupational needs in the hospital or at home after discharge
3. Setting the treatment goals (accomplishment of occupational activity) for each occupational need

Plus, for each goal set:

(4) Definition of the implementation time
(5) Definition of the level of independence expected at the end of the treatment
(6) Planning the appropriate OT intervention (i.e., content and modalities) according to a specific planning checklist (Table 2)

### 2.8. Withdrawal from Trial

Participants were withdrawn from the study for any of the following reasons:

- Serious adverse events or death
- Patient referred to other wards for clinical reasons
- Patient discharged to a nursing home after in-hospital rehabilitation
- Patient lost to follow-up
- Patient withdrawal of consent to participate

All withdrawals with specific reason were recorded. Data collected up to the patient's discontinuation of the study were analyzed with the intention-to-treat approach.

### 2.9. Data Analysis

The analyses were carried out by the Research and Statistics units of the AUSL-IRCCS of Reggio Emilia. This was an exploratory study as there was no information to set the sample size based on statistical criteria. Thus, it was considered appropriate to randomly recruit 40 subjects to estimate the average effect size of experimental OT measured by the performance score of the COPM. To compute the effect size, we compared the changes in COPM performance score between IG and CG in the T2-T0 time frame. Furthermore, to evaluate the clinical relevance of this finding, we matched it with the minimal clinically important difference of the COPM performance score, which was estimated equal to two points [18].

In addition, to estimate the effects of experimental OT in this population, the mean variations of all the outcome measures were compared between groups at the T1-T0, T2-T1, and T2-T0 time frames.

Descriptive statistics were performed to investigate the sample characteristics; mean and standard deviation were chosen to summarize continuous variables, while absolute and relative frequencies (*n*, %) were used for categorical variables.

The assumption of normality for continuous variables was verified statistically using the Shapiro-Wilk test.

To test differences between the groups, numerical data were compared using the Student *t*-test and Mann–Whitney *U* test, and categorical data were compared using Pearson's chi-squared test or Fisher's exact test. The threshold for statistical significance was set at *p* < 0.05. IBM SPSS Statistics 23 for Windows (SPSS, Chicago, IL) was used for statistical analyses.

## 3. Results

**Figure 1:**
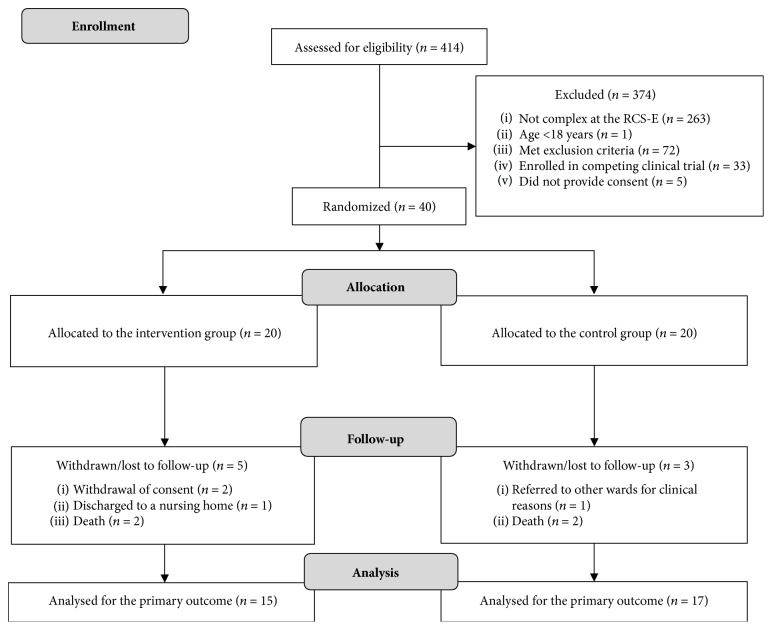
CONSORT 2010 flow diagram of the study.

The Consolidated Standards of Reporting Trials (CONSORT) flow diagram of the RCT is shown in Figure 1. From February 2016 to June 2017, 414 patients were admitted to the PRM ward, 151 of whom were deemed complex. After screening for eligibility, 40 patients were recruited and randomized to experimental intervention or standard care. Eight patients withdrew from the trial and were not reassessed at follow-up (T2), five in the IG, and three in the CG. Two patients verbally withdrew their consent to continue the experimental OT after discharge to their domicile. One participant was discharged to a nursing home, so the home-based phase of experimental OT could not be implemented. One patient dropped out due to clinical worsening during the in-hospital phase, and four participants died.

The baseline demographics and clinical data of the sample are shown in Table 3.

The mean age of all participants was 64 years (range 26–85). Most patients were men (23; 57%). The mean complexity of care needs for the sample was 10.75 (range 9–13), and the average comorbidity index was 5.18 (SD 2.27). At T0, the groups were balanced in terms of the main demographic and clinical characteristics recorded. The diagnoses of access to rehabilitation were acute polyneuropathy (*n* = 11), stroke (*n* = 9), cancer (*n* = 7), hemiparesis due to acute neurological syndrome (*n* = 4), lower limb amputation (*n* = 4), orthopedic diseases or musculoskeletal trauma (*n* = 3), and paraplegia (*n* = 2). The average duration of hospitalization was 48.84 (SD 27.59) days in IG and 40.61 (SD 24.88) days in CG (*p* = 0.34).

**Figure 2:**
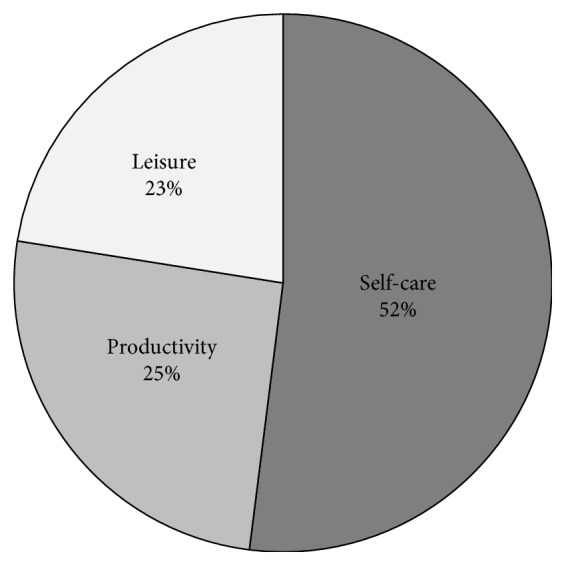
Distribution of relevant occupational activities chosen by participants in this study.

Figure 2 shows the distribution of relevant occupational activities chosen by study participants to satisfy their priority occupational needs and corresponding to the goals of the experimental OT intervention. As expected, most of these activities regarded the self-care area, with particular attention to personal care (32.5%) and functional mobility issues (12%). Within the productivity and leisure areas, household management and dynamic recreation activities were chosen by 14.5% and 14% of participants, respectively.

Regarding the main objective of this study, 32 patients were included in the primary outcome analysis of COPM performance because both baseline and follow-up data were available. The difference between follow-up and baseline scores was, on average, 5.62 (±2.10) for the IG and 2.56 (±1.89) for the CG. Thus, the gain obtained by the experimental OT intervention was significantly higher than by standard care (*p* < 0.001; CI = −4.50 to −1.61) and amply exceeded the MCID established for the COPM performance score (Table 4).

This result was consistent with that of COPM satisfaction: in the same time frame, this difference was on average 5.52 (±2.10) for the IG and 2.70 (±1.88) for the CG. Once again, the gain obtained with the experimental OT intervention was significantly higher than with standard care (*p* < 0.001; CI = −4.25 to −1.37), and again, it was clinically relevant.

To note, the advantage in favor of the intervention group was already evident for COPM performance and satisfaction scores in the T1-T0 comparison.

**Figure 3:**
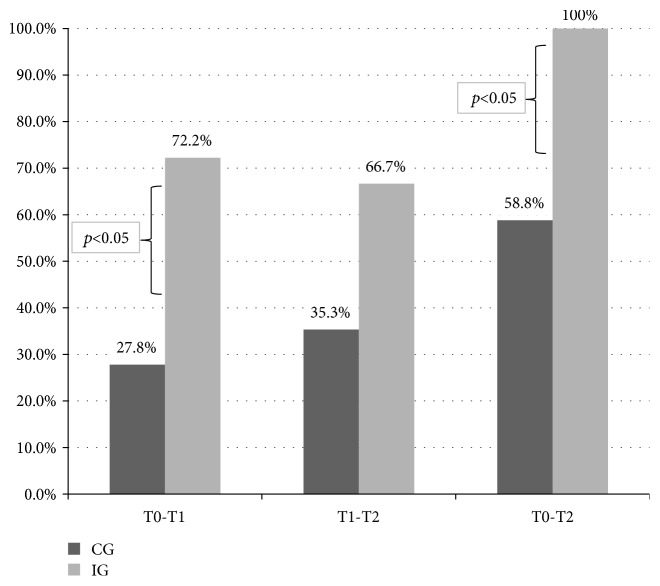
Proportion of patients whose gain in COPM performance score exceeded the minimal clinically important difference set for the COPM.

**Figure 4:**
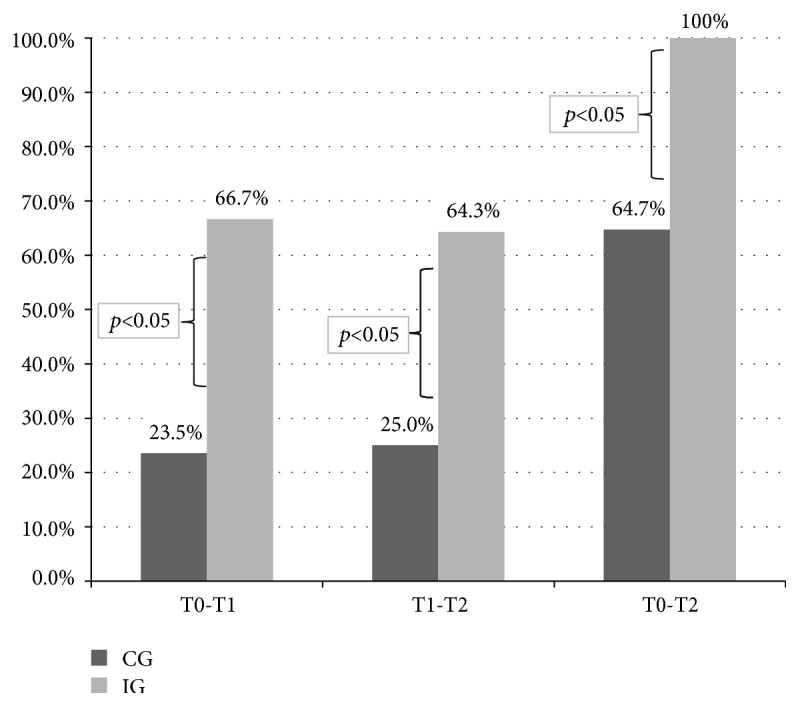
Proportion of patients whose gain in COPM satisfaction score exceeded the minimal clinically important difference set for the COPM.

Figures 3 and 4 show the proportion of patients in the two groups whose gain in COPM performance and satisfaction exceeded the MCID; of note, this proportion was statistically significant for both measures in the comparisons versus baseline. Gain in satisfaction was also significant in the follow-up versus discharge (T2-T1) comparison. Furthermore, at the end of the study, all patients in the IG reached clinically relevant gains in their performance and satisfaction.

Additionally, the change in the IADL score followed a trend consistent with that showed by the COPM.

Concerning the other secondary outcome measures, no other statistically significant between-group differences were demonstrated, although the change in the MBI at the T2-T0 comparison and the change in the RNLI at the T1-T0 comparison were on the verge of statistical significance, in favor of the IG (HADS and SF-12 values are not represented in Table 4).

For completeness of results, Table 5 shows the between-group comparisons of the average scores for each of the clinical outcomes measured at each point in time.

The groups were balanced at baseline, although for the main outcome measure IG scores were clearly less favorable. Despite this, and in line with the results shown in Table 4, the IG demonstrated significantly higher average values for COPM performance and satisfaction at T1 and, above all, at T2. No other significant differences were registered, with the exception of a higher reintegration to normal living scores for the IG at T2.

### 3.1. Feasibility of the OT Experimental Intervention

Fifteen of the 20 complex patients enrolled in the IG (75%) completed the experimental intervention according to the predefined posology. The experimental OT was therefore deemed feasible in the context of application. Furthermore, 70% of participants enrolled in IG achieved at least four treatment goals within the a priori estimated time of implementation and the same percentage reached the goals with the estimated level of independence.

## 4. Discussion

One of the key findings of this pilot study was that the experimental OT intervention, implemented in a mixed hospital-home-based setting, was feasible in complex patients undergoing rehabilitation. Of note, although we did not define a priori harms (e.g., accidental falls) or unintended effects of experimental treatment, none occurred during the course of the study. The results of this study can therefore be generalized in highly complex patients, which are increasingly present in clinical settings.

Moreover, the experimental OT improved patients' performance and satisfaction during the implementation of relevant activities, as perceived by participants. The estimated size effect is undoubtedly clinically relevant, and it may be used in the near future to plan methodologically sound clinical trials.

Further, the experimental OT improved the patients' independence in IADL and enhanced their level of reintegration to normal social activities.

Although current guidelines already state the effects of OT in specific populations [19], this pilot study is the first to confirm that a client-centered OT program based on sound methodology [17] may benefit a population of complex patients, regardless of their underlying disease.

This study also highlights that complex patients would like to be able to satisfy a broad range of needs related to their well-being right from the beginning of rehabilitation, shortly after the onset of an acute illness. These needs range from basic personal-care needs, which many in the population of interest expressed, to more “advanced” needs, which reflected various necessities expressed by only few patients. Therefore, this research also focused on the patients' perspective, oriented towards the return to the community and to social participation right from the beginning of the rehabilitation process, even in the presence of limitations and priority clinical needs. Thus, this study's results encourage the implementation of client-centered OT interventions focused on ameliorating the transition between rehabilitation programs and the community, as also suggested by individuals with long-term physical disabilities [20]. Accordingly, the experimental intervention setting of this study was hospital plus home-based. It is known that the provision of timely home-based rehabilitation facilitates hospital discharge, reduces the risk of readmission, improves functional independence, and helps people with stroke recover social participation [21–23]. Moreover, home-based OT has been proven to improve occupational performance in older adults [24, 25]. Consistent with previous evidence, this study showed an average higher reintegration to normal living of patients treated with experimental OT, compared to the control group, although this advantage was barely significant, and this significance was not confirmed in the between-group differences of average changes for this measure. This might be explained by the fact that reintegration to normal living was addressed in the home-based phase of the intervention, which might not have been of sufficient intensity or duration to maintain the pace of improvement shown in the hospital [21, 24]. In fact, it is evident that the greater gains in all outcomes measured were not obtained in the home-based phase of the study (Table 4), although the experimental OT allowed for slight improvement at follow-up of the positive achievements already obtained at hospital discharge.

A possible limitation of this study is that we excluded patients with cognitive impairment as determined through clinical evaluation. However, we excluded only those patients with severe cognitive impairment, and the intervention performed, which requires good patient compliance, was feasible in the population of interest. It is unlikely that differences in the cognitive level of the patients may have biased the results of the study, since the two groups were balanced for average age and diagnoses represented. However, future studies aimed at verifying the OT effectiveness in a complex and heterogeneous population should also include an objective assessment of the patients' cognitive level.

This study failed to demonstrate a positive effect of OT in important clinical outcome measures such as HADS, QoL, and MBI. Some of these unsuccessful results are consistent with those already shown in patients with stroke, brain tumor, or trauma, where individualized rehabilitation interventions have failed to reduce mood symptoms [26, 27] and QoL [26, 28, 29]. This failure could be due to the limited sample recruited; looking at the results, it should be noted that at baseline, the IG scores were worse than those of the CG in all the outcomes measured, though not statistically significantly so. As during the study the gain obtained by the IG was definitely greater than that obtained by the CG, it is plausible that a larger sample would have had the power to highlight this difference even in statistical terms. However, it should not be forgotten that the study was designed with the main objective of detecting the effect size of OT on the performance score of the COPM, and this aim was completely achieved. Nevertheless, when interpreting the size of the estimated OT effect, the fact that the COPM was administered by an occupational therapist not blinded to the allocation group of patients and involved in the experimental treatment must be borne in mind. This limit may thus have biased the results of this study. The choice of administering the COPM open label was dictated by local constraints in professional and environmental resources, which themselves would have prevented the blindness of the assessments. However, to the best of our knowledge, the COPM is not usually administered blindly because, by its nature, it entails the building of a partnership between patient and assessor that constitutes the foundation for OT interventions. We suggest that future studies combine COPM with blinded measures of relevant outcomes.

## 5. Conclusions

To conclude, this pilot study allowed the detection of the effect size of experimental OT implemented in complex patients in a hospital-home-based setting. This effect size was markedly greater than the MCID detected for the COPM, a client-centered outcome measure widely used in clinical and research settings. Thus, this study strongly supports the application of client-centered rehabilitation programs supplemented by OT right from the early stages of rehabilitation of patients with high clinical complexity levels. Moreover, the effect size detected in this research study will help scientists design future powered randomized controlled trials aimed at confirming the effectiveness of client-centered OT in similar populations and heterogeneous settings.

## Figures and Tables

**Table 1:** Clinical outcome measures and study assessments.

	T0 Baseline (within one week from admission to PRM ward)		T1 Discharge (within 3 days up to discharge)	T2 Follow up (45 ± 15 days from discharge)
Comorbidities (CCI)	X	Randomization		
Performance in carrying out occupational activities (COPM)	X		X	X
Satisfaction in carrying out occupational activities (COPM)	X		X	X
Mood disturbances (HADS)	X		X	X
B-ADL (MBI)	X		X	X
I-ADL (IADL)	X		X	X
Reintegration to Normal Living Index (RNLI)			X	X
Quality of life (SF-12)			X	X

PRM: physical and rehabilitation medicine; CCI: Charlson Comorbidity Index; COPM: Canadian Occupational Performance Measure; HADS: Hospital Anxiety and Depression Scale; B-ADL: basic activities of daily living; MBI: modified Barthel Index; I-ADL: instrumental activities of daily living; IADL: instrumental activities of daily living; RNLI: Reintegration to Normal Living Index; SF-12: Short Form-12.

**Table 2:** Planning checklist of OT intervention for each goal set.

Phase	Activity	Options	
A	Definition of the OT rehabilitative approach	Restorative	Compensative
B	Definition of treatment posology	Number of sessions	
		Frequency of sessions	
		Duration of each session	
C	Definition of any supports and/or facilitation strategies to be used during sessions (e.g., aids, caregivers, and facilities)		
D	Definition of the intervention setting/s		
E	Group sessions	Yes	No

**Table 3:** Baseline characteristics of the study participants.

Characteristics	IG (*n* = 20)	CG (*n* = 20)	*p* value
Age (mean ± SD)	66.35 (±14.67)	62.75 (±10.99)	0.38^a^
Gender, F/M (%)	8/12 (40/60%)	9/11 (45/55%)	0.74^b^
RCS-E (mean ± SD)	10.75 (±0.96)	10.75 (±0.91)	1.00^a^
CCI (mean ± SD)	5.20 (±2.41)	5.15 (±2.18)	0.94^a^
Main diagnosis of access for rehabilitation (%)			0.853^b^
Acute polyneuropathy	6 (30%)	5 (25%)	
Stroke	5 (25%)	4 (20%)	
Cancer	4 (20%)	3 (15%)	
Hemiparesis due to acute neurological syndrome	2 (10%)	2 (10%)	
Lower limb amputation	1 (5%)	3 (15%)	
Orthopedic diseases or musculoskeletal trauma	1 (5%)	2 (10%)	
Paraplegia	1 (5%)	1 (5%)	

IG: intervention group; CG: control group; SD: standard deviation; ^a^Student's *t*-test; F/M: female versus male ratio; ^b^Pearson's chi-square test or Fisher's exact test; RCS-E: Rehabilitation Complexity Scale-Extended; CCI: Charlson Comorbidity Index.

**Table 4:** Between-group differences of average changes during the study course for the main clinical outcome measures.

	ΔIG (mean ± SD)	ΔCG (mean ± SD)	*p* value	Mean difference (95% CI)
COPM perf.				
T1-T0	3.18 ± 1.89	1.21 ± 1.13	0.001	−1.97 (−3.03; −0.91)
T1-T2	1.98 ± 1.98	1.26 ± 2.16	0.336	−0.72 (−2.22; 0.78)
T2-T0	5.62 ± 2.10	2.56 ± 1.89	0.001	−3.06 (−4.50; −1.61)
COPM satisf.				
T1-T0	3.01 ± 2.25	1.26 ± 1.15	0.006	−1.75 (−2.96; −0.53)
T1-T2	1.97 ± 2.53	1.35 ± 2.05	0.454	−0.61 (−2.27; 1.04)
T2-T0	5.52 ± 2.10	2.70 ± 1.88	0.001	−2.81 (−4.25; −1.37)
MBI				
T1-T0	41.77 ± 20.34	30.16 ± 22.37	0.113	−11.61 (−26.10; 2.88)
T1-T2	6.00 ± 6.34	4.52 ± 10.98	0.652	−1.47 (−8.06; 5.12)
T2-T0	49.33 ± 20.14	37.00 ± 18.58	0.082	−12.33 (−26.31; 1.64)
IADL				
T1-T0	−1.15 ± 0.81	−0.51 ± 0.46	0.007	0.64 (0.18; 1.09)
T1-T2	−0.55 ± 0.62	−0.53 ± 0.79	0.938	0.02 (−0.50; 0.54)
T2-T0	−1.83 ± 0.88	−1.06 ± 0.78	0.014	0.76 (0.16; 1.36)
RNLI				
T2-T1	24.46 ± 20.28	10.47 ± 21.39	0.068	−13.98 (−29.05; 1.07)

Δ = within-group change; IG: intervention group; CG: control group; SD: standard deviation; CI: confidence interval; COPM: Canadian Occupational Performance Measure; perf.: performance; satisf.: satisfaction; MBI: modified Barthel Index; IADL: instrumental activities of daily living; RNLI: Reintegration to Normal Living Index.

**Table 5:** Between-group comparisons of average scores for all clinical outcome measures.

	T0			T1			T2		
	IG (*n* = 20)	CG (*n* = 20)	*p* value	IG (*n* = 18)	CG (*n* = 18)	*p* value	IG (*n* = 15)	CG (*n* = 17)	*p* value
COPM perf. (mean ± SD)	1.73 ± 0.78	2.21 ± 0.81	0.064	4.89 ± 1.73	3.44 ± 1.41	0.009	7.28 ± 1.67	4.77 ± 1.89	0.001
COPM satisf. (mean ± SD)	1.86 ± 0.69	2.22 ± 1.05	0.21	4.86 ± 2.0	3.55 ± 1.44	0.031	7.33 ± 1.89	4.98 ± 1.81	0.001
HADS anx. (mean ± SD)	6.90 ± 3.74	6.80 ± 3.79	0.93	5.78 ± 3.93	5.56 ± 4.20	0.87	4.13 ± 3.29	6.77 ± 4.67	0.08
HADS depr. (mean ± SD)	7.40 ± 4.26	6.35 ± 3.94	0.42	6.44 ± 4.33	6.17 ± 4.20	0.85	5.53 ± 3.16	6.65 ± 5.04	0.47
MBI (mean ± SD)	37.85 ± 20.32	49.60 ± 25.11	0.11	77.11 ± 23.29	80.50 ± 20.17	0.64	88.00 ± 14.40	88.18 ± 13.20	0.97
IADL (mean ± SD)	5.09 ± 1.15	4.55 ± 1.12	0.14	3.99 ± 1.26	4.01 ± 0.97	0.95	2.98 ± 1.13	3.42 ± 1.06	0.26
RNLI (mean ± SD)				46.86 ± 17.11	46.81 ± 16.79	0.99	72.57 ± 15.56	58.11 ± 22.04	0.043
SF-12 PCS (mean ± SD)				32.80 ± 5.39	33.89 ± 7.89	0.63	34.84 ± 7.19	31.68 ± 7.49	0.23
SF-12 MCS (mean ± SD)				42.59 ± 11.05	43.21 ± 17.09	0.90	46.32 ± 11.87	42.90 ± 13.41	0.45

IG: intervention group; CG: control group; COPM: Canadian Occupational Performance Measure; perf.: performance; SD: standard deviation; satisf.: satisfaction; HADS: Hospital Anxiety and Depression Scale; anx.: anxiety; depr.: depression; MBI: modified Barthel Index; IADL: instrumental activities of daily living; RNLI: Reintegration to Normal Living Index; SF-12: Short Form-12; PCS: Physical Health Composite Score; MCS: Mental Health Composite Score.

## Data Availability

The demographics and clinical data collected to support the findings of this study are restricted by the Ethics Committee of the Province of Reggio Emilia (Italy) in order to protect patient privacy. Data are available from Dott. Debora Formisano, Scientific Directorate, Arcispedale Santa Maria Nuova-IRCCS, Azienda Unità Sanitaria Locale, Reggio Emilia, 42100, Italy (debora.formisano@ausl.re.it), for researchers who meet the criteria for access to confidential data.
